# Scaling Up AIDS Treatment: What Is the Potential Impact and What Are the Risks?

**DOI:** 10.1371/journal.pmed.0020039

**Published:** 2005-01-11

**Authors:** Peter Lamptey, David Wilson

## Abstract

Lamptey and Wilson discuss the implications of a new study showing that combining treatment with prevention is the best approach to tackling the HIV pandemic.

There has been a recent and dramatic rise in global funding for HIV/AIDS, from US$2.1 billion in 2001 to US$6.1 billion in 2004 [1], thanks to several new funding mechanisms (Box 1). These funds, coupled with reduced drug costs, make it feasible to roll out antiretroviral therapy (ART) even in resource-poor settings. Nevertheless, the total number of people living with HIV rose in 2004 to reach its highest level ever: an estimated 39.4 million people are living with the virus, including 4.9 million who acquired it in 2004 [1]. Therefore, the debate over the appropriate distribution of money between prevention efforts (such as voluntary counseling and testing [VCT], or behavior change) and treatment efforts (the provision of ART) is now more topical than ever.

**Box 1. Initiatives to Fund ART**

- **The WHO “3 by 5” Initiative:** This initiative aims to place 3 million people in low- and middle-income countries on ART by the end of 2005.
- **The US President's Emergency Plan for HIV/AIDS Relief:** This initiative aims to treat 2 million HIV-infected people with ART, to prevent 7 million new infections, and to care for 10 million HIV-infected individuals and AIDS orphans in five years (2004–2009).
- **The Global Fund to Fight AIDS, Tuberculosis, and Malaria:** In the five years following its inception (2002–2007), the Global Fund aims to provide 1.6 million people with ART and 52 million people with VCT, and to support more than 1 million orphans with medical services, education, and community care.

## Balancing Prevention and Treatment

The scale of the proposed increase in the number of patients receiving ART raises numerous questions about the treatment itself. Which drugs will be used? How much will it cost? How will their quality be monitored and assured? How will they be distributed? Who will be eligible? How will the desired level of treatment be sustained? Is there adequate infrastructure and human resources to support the expanded services?

The commitment of substantial funding to treatment in resource-poor countries also has implications for the prevention efforts in those same countries. In many Western countries and Brazil (the sources of the majority of the available data on the subject), the impressive drop in mortality due to HIV following increased access to ART is coupled with a disheartening rise in the number of new cases of HIV, as emphasis and funding are shifted from prevention to treatment [2]. Countries in which this pattern has been seen are evidence of the pitfalls of failing to adapt prevention efforts once life-extending treatment becomes widely available.

Of course, prevention and treatment are not mutually exclusive. Successful prevention efforts mean fewer patients will need the costly drug treatment programs, helping extend the sustainability of ART. In turn, the success of ART in prolonging healthy living helps prevention efforts by reducing the stigma associated with self-education and responsible behaviors.

## Measuring Prevention and Treatment Effects

In their study in the January 2005 issue of *PLoS Medicine*, “Integrating HIV Prevention and Treatment: From Slogans to Impact,” Salomon and colleagues use mathematical modeling to assess the epidemiologic impact of treatment and prevention efforts, and to quantify the opportunities and potential risks of large-scale treatment roll-out. Using a variety of different scenarios, they propose methods for establishing the most effective balance between spending on prevention and spending on treatment.

Modeling is a technique used by many scientists, including epidemiologists and statisticians, to create a mathematical equation that can be used to determine which variables affect an outcome of interest, and to what extent. Once the influential variables are determined, a baseline model is established that includes those variables and reflects their relative importance to the outcome. The effect of changing the value of any of these variables, or several of them, can then be tested, and new outcomes projected. HIV modeling is inexact and requires far better data but can nevertheless provide important insights.

Salomon and colleagues used mathematical modeling to assess the effect of changing aspects of the HIV/AIDS “equation” on the future course of the HIV/AIDS epidemic. First, a baseline model was created to fit expected HIV/AIDS projections for the year 2020 if there were to be no change in the current epidemiologic trends—no ART scale-up, and no changes in prevention efforts or behavior. Heterosexual contact is the predominant mode of HIV transmission across Africa, and Salomon and colleagues' study modeled the disease only within the heterosexual population. The model was also tailored to take into account epidemiologic, demographic, and sociologic patterns in the eastern, central/western and southern regions of Africa. Using the baseline models tailored to each region, the effects of prevention and treatment efforts were then measured.

Two treatment-centered scenarios were tested in which the World Health Organization's “3 by 5” initiative (see Box 1) was achieved. In these treatment-centered scenarios, the reduction of transmissibility, the number of partners of each patient, and condom use were either optimal (reduced transmissibility, reduced partners, and increased condom use) or less than optimal. The prevention-centered scenario tested the impact of a comprehensive package of 12 prevention tools (such as VCT and peer counseling for sex workers), modeling only partial effectiveness at the population level, to reflect weaker political and social support for HIV control efforts. Finally, combined response scenarios were tested. In the first scenario, treatment efforts strengthened prevention efforts as, for example, when the availability of ART increases people's willingness to undergo testing. In the second, an emphasis on treatment led to less effective implementation of prevention efforts.

Baseline projections in Salomon and colleagues' study showed that without any behavioral change or ART scale-up, the HIV/AIDS prevalence rate would remain relatively stable, but the number of new infections would increase by 52.3 million by 2020. Treatment-centered scenarios reduced the total number of new infections through 2020 by a maximum of 3 million, or 6%, while indicating that the number of AIDS deaths through 2020 would decline by 13%, to 32.4 million. A prevention-centered strategy would provide greater reductions in incidence (36%) and similar mortality reductions by 2020, but more modest mortality benefits over the next five to ten years.

**Figure 1:**
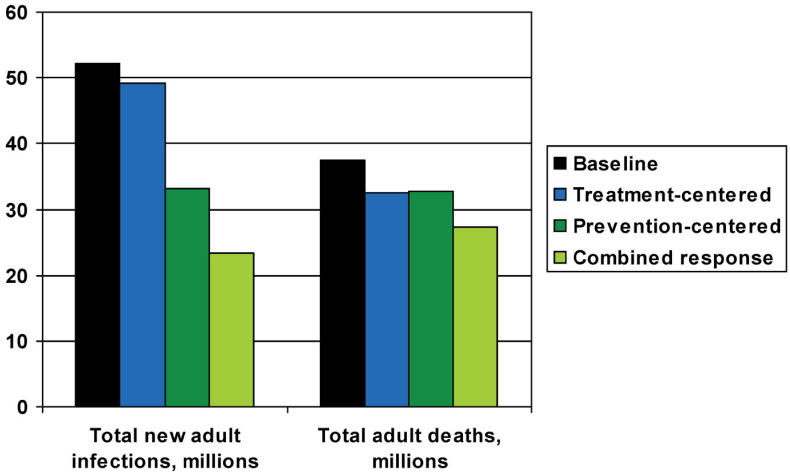
Projected New Adult Infections and Total Adult Deaths, in Millions, to 2020 This graph represents projections through 2020, and, when there was a choice, highlights the more successful iteration of a model. The treatment-centered response, therefore, shows data from the optimal-effects model, and the combined response data reflect the optimistic model.

The scenarios in which all of these statistics were most improved, however, were those that combined both prevention and treatment efforts. In the scenario in which treatment enhanced prevention, Salomon and colleagues projected 29 million averted infections (55%) and 10 million averted deaths (27%) through the year 2020. However, if a narrow focus on treatment scale-up leads to reduced effectiveness of prevention efforts, the benefits of a combined response would be considerably smaller—9 million averted infections (17%) and 6 million averted deaths (16%) (Figure 1).

Combining treatment with effective prevention efforts could reduce the resource needs for treatment dramatically in the long term. In the various scenarios the numbers of people being treated in 2020 ranges from 9.2 million in a treatment-only scenario with mixed effects, to 4.2 million in a combined response with positive treatment–prevention synergies.

## Moving Forward

**Figure 2:**
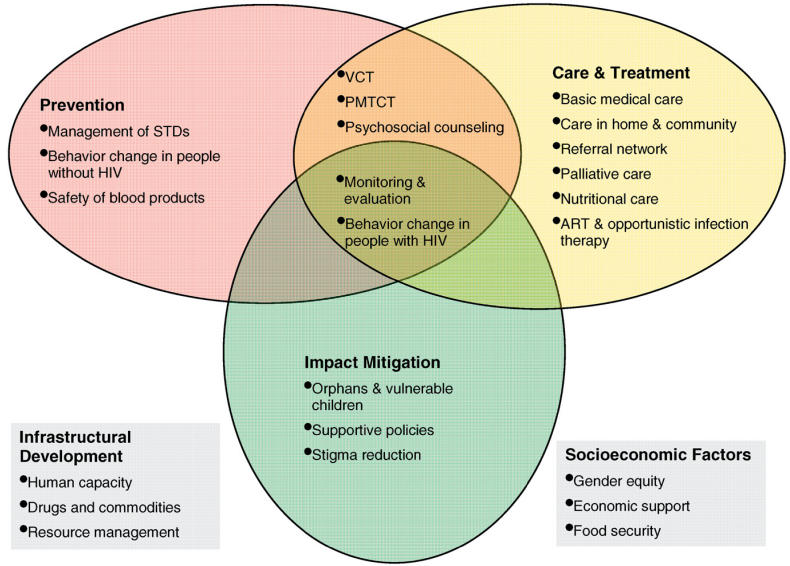
Components of an Integrated Comprehensive HIV/AIDS Program PMTCT, prevention of mother-to-child transmission; STD, sexually transmitted disease.

The authors have demonstrated through mathematical modeling that the integration of treatment and prevention is epidemiologically sound. However, an integrated and comprehensive program (Figure 2) is not only logical but makes sense from the service delivery point of view: it can be cost-effective and ideal for the community.

**Effective prevention makes treatment more affordable and sustainable.** Effective prevention can lead to a substantial reduction in the number of new infections and therefore ultimately will lead to a reduction in the number of people who will need treatment. The reduction of adult HIV/AIDS prevalence in Uganda from 18.5% to 6% over the last several years has reduced the number of those eventually needing treatment by nearly 68% [3]. Unless the incidence of HIV is sharply reduced, HIV treatment will not be able to keep pace with all those who will need therapy [4]. Salomon and colleagues' reaffirmation that only effective prevention will make treatment affordable is critically important.

**Successful treatment and care can make prevention more acceptable and effective.** Widespread access to treatment could bring millions of people into health-care settings, providing new opportunities for health-care workers to deliver and reinforce HIV prevention messages and interventions [4]. Improved access to HIV testing provides an entry point to both prevention and treatment services and provides a unique opportunity to identify and target the infected, vulnerable, and uninfected with more appropriate interventions. All health-care settings, including HIV treatment sites, should deliver HIV prevention services [4].

**Prevention can make treatment more accessible.** The early establishment of community-based prevention services in rural Ghana was instrumental in reducing the stigma of AIDS and improving the knowledge and attitude of the community prior to the development of ART and VCT services (K. Torpey, personal communication). This process also made it easier for community and implementing agencies to identify and refer patients needing treatment services.

**Expanded care and prevention activities have synergistic effects.** Continued effective treatment, care, and prevention programs will reduce the number of orphans and vulnerable children, reduce mother to child transmission of the virus, and improve the lives of families and the strength of communities.

**Integration ensures that prevention activities are not neglected.** The world has a unique opportunity, as ART services are launched and expanded, to simultaneously bolster prevention efforts [4]. Experience in the United States indicates that availability of treatment can lead to increased risk behavior [5]. In addition, the improvement in the health, well-being, and longevity of people living with AIDS could increase the opportunities for HIV transmission. Integration can help reduce these potential negative impacts of treatment.

**Integration can provide opportunities to address vulnerable groups more effectively.** A commitment to providing large-scale treatment helps to focus attention on communities at greatest risk, particularly in lower prevalence contexts. This provides an opportunity to address the prevention and treatment needs of vulnerable groups more effectively.

**Treatment resources can help improve infrastructure for prevention and other health services.** The training of health providers and improvements in laboratory services, pharmacy, logistics, commodity management, and health information systems can benefit both treatment and prevention services. Further, in many countries, a large number of health-care workers are themselves infected. Treatment can help to preserve the lives and productivity of these critically needed AIDS prevention and treatment workers, as well as those of other health professionals.

**A long-term decline in AIDS deaths may be preventing new infections.** The short-term decline in AIDS deaths is driven by effective care and treatment programs, but a long-term decline may be driven by the prevention of new infections. Integrated and comprehensive strategies are more likely to lead to affordable, sustainable programs.

**Success requires dramatic expansion of both ART and prevention.** Globally, fewer than one in five people at high risk of infection have access to proven HIV prevention interventions [6] and less than 10% have access to ART [1]. Unless there is a substantial increase in commitment and resources for both prevention and ART, efforts to control HIV/AIDS and mitigate its impact will only meet with partial and limited success. In addition, to increase resources, intensified commitment is required to ensure every opportunity is taken to integrate prevention and treatment. Future analysis and debate should move from comparisons of prevention and treatment priorities to a sustained analysis of how we can reciprocally integrate and strengthen prevention and care and use every opportunity provided by one to reinforce the other. We must focus on the development of training, monitoring, and quality assurance systems that ensure that prevention and care are integrated whenever possible.

**The results of Salomon and colleagues' model need to be validated.** Further operational research is needed to validate the findings of this study.

## Abbreviations

ART: antiretroviral therapy
VCT: voluntary counseling and testing
